# Generation Times of *E. coli* Prolong with Increasing Tannin Concentration while the Lag Phase Extends Exponentially

**DOI:** 10.3390/plants9121680

**Published:** 2020-12-01

**Authors:** Sara Štumpf, Gregor Hostnik, Mateja Primožič, Maja Leitgeb, Urban Bren

**Affiliations:** 1 Faculty of Chemistry and Chemical Engineering, University of Maribor, Maribor 2000, Slovenia; sara.stumpf@um.si (S.Š.); gregor.hostnik@um.si (G.H.); mateja.primozic@um.si (M.P.); maja.leitgeb@um.si (M.L.); 2 Faculty of Medicine, University of Maribor, Maribor 2000, Slovenia; 3 Faculty of Mathematics, Natural Sciences and Information Technologies, University of Primorska, Koper 6000, Slovenia

**Keywords:** antimicrobial activity, growth phases, growth rate, growth medium, natural products

## Abstract

The current study examines the effect of tannins and tannin extracts on the lag phase duration, growth rate, and generation time of *Escherichia coli*. Effects of castalagin, vescalagin, gallic acid, Colistizer, tannic acid as well as chestnut, mimosa, and quebracho extracts were determined on *E. coli*’s growth phases using the broth microdilution method and obtained by turbidimetric measurements. *E. coli* responds to the stress caused by the investigated antimicrobial agents with reduced growth rates, longer generation times, and extended lag phases. Prolongation of the lag phase was relatively small at low tannin concentrations, while it became more pronounced at concentrations above half the MIC. Moreover, for the first time, it was observed that lag time extensions follow a strict exponential relationship with increasing tannin concentrations. This feature is very likely a direct consequence of the tannin complexation of certain essential ions from the growth medium, making them unavailable to *E. coli* for its growth.

## 1. Introduction

Numerous plants with medicinal effects can be found all over the world. Following the discovery of antibiotics, plant derivatives were hardly used as antimicrobial agents. After the overprescription and misuse of traditional antibiotics caused the rise of antimicrobial resistance, various new sources, especially plants, are being investigated for potential antimicrobial agents [1,2]. Numerous plant extracts and their purified substances, e.g., tannins, were identified as suitable alternatives for antibiotics [3,4].

Tannins represent water-soluble polyphenols that are found in numerous plants. They possess the ability called astringency, which enables them to precipitate proteins, which differentiates them from other phenolic compounds [5]. Tannins are typically classified into hydrolysable and condensed tannins [6]. Hydrolysable tannins represent esters of phenolic acids and polyols, e.g., glucose. Depending on the type of phenolic acids, they are divided into gallotannins, which can be hydrolyzed to gallic acid, and ellagitannins, which can be hydrolyzed to ellagic acid. Proanthocyanidins, also known as condensed tannins, got their name from their feature that when heated in acidic media, they produce anthocyanidins. They represent polymers composed of flavan-3-ol units and they usually possess a higher molecular weight than hydrolysable tannins [7,8]. Commercial tannins are usually plant extracts, composed of a mixture of gallotannins (e.g., tannic acid), ellagitannins (e.g., chestnut extract), or condensed tannins (e.g., quebracho extract) [9]. Their primary application was tanning of animal skins [9]. They exert several beneficial health effects like antiseptic, anticarcinogenic, anti-inflammatory activity [3,10,11], and antibacterial activity [12,13] which makes them also suitable for pharmaceutical and nutraceutical applications. Moreover, they play an important role as a raw material for sustainable green industries [14].

**Figure 1:**
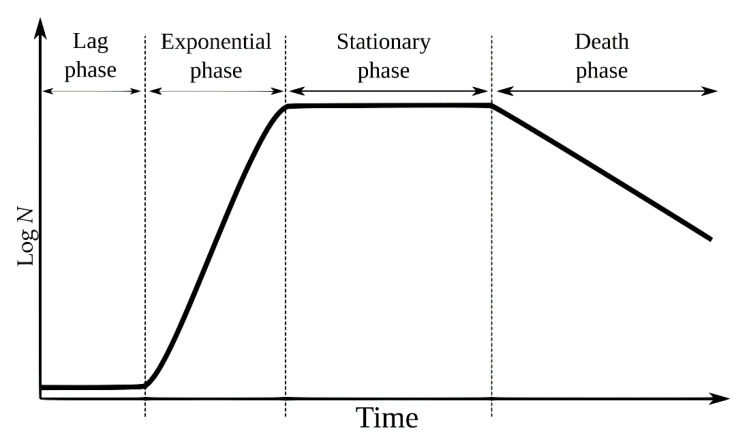
A typical microbial growth curve in a closed system, where *N* represents the number of bacterial cells.

Microorganisms are largely present in our surrounding and live in almost every habitat. They affect the everyday life of humans in both beneficial and detrimental ways. Bacterial growth is represented with a growth curve, which illustrates the growth of the bacterial population in a closed system. The growth curve consists of four phases, as depicted in Figure 1.

The first phase represents the lag phase (λ), which lasts from the inoculation of bacteria until the growth of the bacterial population. During this time, bacteria increase in cell size, but do not divide [15]. It fits the adaptation period in which bacteria adapt to the new environment and processes such as biosynthesis of various essential constituents, RNA transcription, accumulation of different metals (i.e., iron, calcium, manganese), and Fe-S cluster formation, that are necessary for the growth of the population [16]. The duration of the lag phase is influenced by the inoculum size, the physiological history of the cells, and the physiochemical environment of the original and of the new growth medium. The next phase represents the exponential phase in which the cell population doubles at regular intervals according to different growth rates (μ). The time at which the bacterial population doubles is known as doubling or generation time (*t*_d_). Generation times depend on incubation conditions and the bacterial organism itself and can last from few minutes to few days. When the generation time is known, the number of bacterial cells after this time (*N*) can be obtained according to the expression:

(1) $$\begin{array}{c} N=N_{0}\times 2^{n} \end{array}$$

where *N*_0_ is the starting number of bacterial cells and *n* is the number of generations. How the number of bacterial cells changes over time can also be calculated from the expression:

(2) $$\begin{array}{c} N=N_{0}e^{µt} \end{array}$$

where *μ* represents the growth rate (d^−1^, h^−1^ or min^−1^) and *t* is time (d, h or min). The exponential phase is followed by a stationary phase, where the number of new cells in every time interval equals the number of cells that die in the same time interval. When the nutrients in the growth medium are exhausted, the bacterial population starts to diminish. This final phase is called the death phase [15].

Pathogens are microorganisms that can cause diseases in their hosts [15]. Among the many pathogenic bacteria responsible for numerous infectious diseases that pose a real threat to public health, one also finds strains of Gram-negative bacteria *Escherichia coli*, which are producing enterotoxins [17]. *E. coli* also represents one of the most studied microorganisms. Several methods are used to determine the in vitro susceptibility of microorganisms to antimicrobials. The most widely applied methods include broth dilution and disc or agar well diffusion assays. Diffusion methods are used to provide qualitative or even semi-quantitative results on whether microorganisms are susceptible or resistant to the assayed antimicrobial agent. On the other hand, it is possible to quantitatively determine the antimicrobial activity of studied agents by determining the minimum inhibitory concentration (MIC) and minimum bactericidal concentration (MBC) using dilution methods [15,18,19].

MIC values of several antimicrobial agents have already been examined, some against *E. coli*, others against different microorganisms [20,21,22,23,24,25,26,27,28,29]. Although there are several studies available, a comparison between determined MIC values is difficult since the outcome of an assay is affected by many factors, including the bacterial strain [24] and growth medium used [30], inoculum concentration [31], incubation time and temperature [30] as well as the employed method of MIC determination [32]. In our previous study, MIC values of tannins and tannin extracts were determined while we were also able to show that these values increase linearly with the growth media strength [33]. One can also find studies on the effect of antimicrobials on bacteria below the MIC. The first change made by bacteria in response to antibiotic stress occurs in the lag phase in order to develop the tolerance to antibiotics. Consequently, bacteria can survive in the environment with high antibiotic concentrations, and this may initiate the evolution of antibiotic resistance in bacteria. Thus, it can be concluded that the lag phase represents the key step in the development of antibiotic resistance strategies, so an in-depth understanding of how antimicrobials affect the lag phase is essential for a reliable assessment of the antibacterial resistance [34]. Extended lag phases and decreasing growth rates have already been observed for antibiotics in bacteria [35,36], as well as for certain tannins, including tannic acid, in different species of *Streptococcus* [37].

The determination of MIC is usually applied as a standard method to indicate the resistance of a microorganism to antimicrobial agents. Although MICs indicate the inhibition of bacterial growth, they provide only limited information about resistance mechanisms. The lag phase, however, remains poorly understood due to the low metabolic rate of cells and, consequently, there are not many data published on the effects of tannins on lag and generation times. A novel advanced approach to monitoring the bacterial stress response, allowing for both quantitative and mechanistic evaluation of the bacterial resistance against antimicrobial agents, represents the determination of lag time extension (LE). Evaluating the antimicrobial resistance of bacteria without considering the LE can lead to erroneous findings, which in clinical cases, can even result in failed treatment. Therefore, the present study is aimed at examining the effects of pure tannins as well as tannin extracts on the growth kinetics of *E. coli* by determining generation times and the extension of lag phases. Bacterial growth was followed using the broth microdilution method by monitoring turbidity every 5 min for 20 h.

## 2. Results and Discussion

### 2.1. Growth Rates and Generation Times

Concentrations of tested agents were selected in the way that at first preliminary experiments with twofold dilutions of the agents were performed to determine approximate MIC values for each agent. Thereafter, more precise MIC values were determined by measuring the turbidity of seven dense concentrations close to the MIC determined before.

Using turbidimetric method, lag phase durations and bacterial growth rates were obtained. Semilogarithmic graphs of the logarithm of OD as a function of time were drawn. From the semilogarithmic graphs, the growth rates were determined. As can be observed from Table 1, growth rates of *E. coli* for all concentrations of all tested agents were lower than that of the negative control and were mostly decreasing with higher concentrations of the investigated agents. It should be noted that this trend was not always very pronounced and that there were several fluctuations. These fluctuations can be attributed to the experimental uncertainty that occurs due to the growth rate determination method. The variability in the experimental points already contributes to it, and the selection of the linear area represents an even greater contributor since the growth curves are rarely completely in line with the theoretically predicted ones.

The generally decreasing trend of *E. coli* growth rates was observed for all antimicrobial agents except for Colistizer. This trend was the most prominent for castalagin and gallic acid as well as chestnut, quebracho, and mimosa extracts. For other agents, this trend was not that obvious, for example, the tannic acid growth rate at 40 µg/mL stood out while for other concentrations growth rates were likewise decreasing. For lower concentrations of vescalagin, the decreasing trend was also observed, but at higher concentrations the growth rate was constant. Growth rates of *E. coli* with added Colistizer remained almost unaffected at all concentrations. The differences in the growth rates could be partially attributed to the fact that the tested samples predominantly comprised of crude extracts with varying composition of different compounds, which can considerably affect the growth rates.

Generation times were calculated from growth rates using Equation (3). Literature states that a generation time of *E. coli* at optimal conditions amount to 20 min [38], which corresponds to the generation time of the negative control observed in this study. As expected, generation times were mostly increasing with increased tannin concentrations. However, this trend was not very pronounced due to the experimental uncertainty in the determination of generation times, as evident from Equation (3) and the discussion about growth rates. The trend of prolonged generation times with increased antimicrobial agent concentrations was the most prominent in the case of castalagin and gallic acid as well as chestnut, quebracho, and mimosa extracts. This trend was also preserved for tannic acid, although there was one outlier. In the case of vescalagin, generation times were increasing at lower concentrations, while at higher concentrations the generation times became stagnant. The biggest deviation from this trend was the generation times of *E. coli* in the case of Colistizer, which varied very little between the tested concentrations.

All in all, with increasing tannin concentrations, the growth rates of *E. coli* were generally decreasing, while generation times were on average prolonged. This result was expected, since the proposed molecular mechanisms of tannin antimicrobial activity include [9] (a) interaction of tannins with bacterial and growth medium proteins; (b) interaction of tannins with bacterial cell wall plasma membrane; and (c) chelation of metal ions, which all result in the addition of tannins producing less favorable conditions in growth media for bacterial growth.

### 2.2. Lag Times

**Figure 2:**
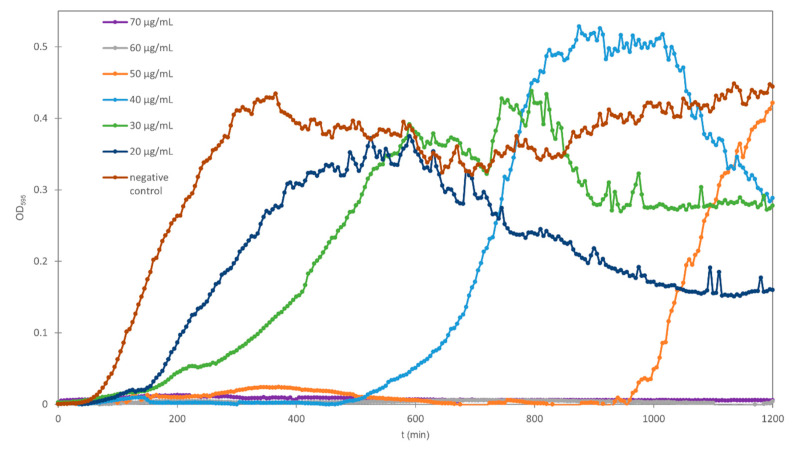
Growth curves of *E. coli* for negative control and for different concentrations of tannic acid (time dependence of OD at wavelength of 595 nm).

Growth curves plotting the time dependence of OD at wavelength of 595 nm were constructed. Table 1 reports lag phase durations besides growth rates and generation times. Prolonged lag times of *E. coli* for all concentrations of all investigated antimicrobial agents were observed compared to the negative control assay. Lag times were prolonged with increasing concentrations of the investigated agent; however, this trend was not equally prominent for all tannins. An example of the experimentally obtained *E. coli* growth curves for negative control and for different concentrations of tannic acid is presented in Figure 2, where the trend of lag phase prolongation with increasing tannin concentration was clearly observed.

**Figure 3:**
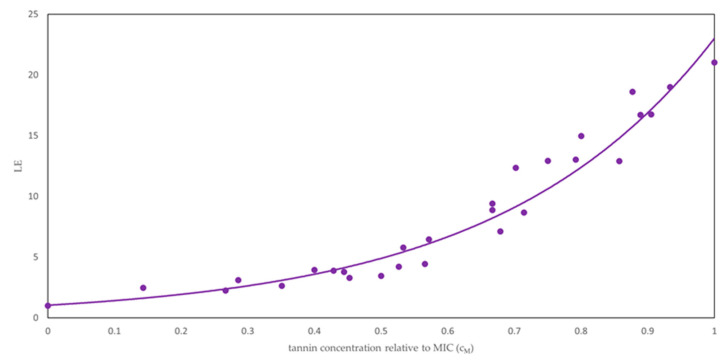
Dependence of lag time extension (LE) on the normalized concentration of tested agents vescalagin, castalagin, tannic acid, Colistizer as well as the quebracho and mimosa extracts ($c_{M}$).

Mean MIC values of pure tannin compounds gallic acid, castalagin and vescalagin as well as of Colistizer, tannic acid, chestnut, quebracho, and mimosa tannin extracts against *E. coli* determined by OD measurements were reported in our previous study [33]. It was observed that MIC values vary significantly between different tannins, with tannic acid being the best inhibitor of the *E. coli* growth (MIC = 57 µg/mL) and gallic acid representing the least effective inhibitor (MIC = 950 µg/mL). Since MIC values of various tannins were very different and consequently the comparison of lag phase duration was difficult, lag time extension (LE) calculated using Equation (4) was plotted against the normalized concentration of examined agents ($c_{M},\;$see Equation (5)) as depicted in Supplementary Material Figure S1. It was observed that the obtained data could offer two interpretations. The first interpretation is that, for all tested samples at concentrations of agents below half the MIC, the lag phase of *E. coli* was only slightly prolonged. However, at tannin concentrations larger than half the MIC, the lag times were greatly extended. The second interpretation of the observed trend is that the lag time extends exponentially with the increase in tannin concentration. The exponential function was fitted to the experimental points and the results are presented graphically in Supplementary Material Figures S2–S9 for each investigated agent individually. The obtained exponential functions with corresponding correlation coefficients (*R*^2^) are presented in Supplementary Material Table S1. It can be observed that the exponential function fits the LE values for all investigated agents very well (*R*^2^ = 0.965–0.998). The only exception represents the fitting of the exponential function to the data for gallic acid, where a somewhat lower agreement between the experimental points and the fitted function was obtained (*R*^2^ = 0.893). Therefore, it can be clearly stated that LE increases exponentially with the rising concentration of investigated tannins. Moreover, if we plot all data points on the same diagram (Supplementary Material Figure S1), we can observe that values for a great majority of agents are roughly the same (they follow the same exponential curve). Only two outliers can be identified, the LE values for the growth of *E. coli* with added chestnut extract (composed mostly of gallotannins and gallic acid) or gallic acid. We believe that their lower values might be a consequence of a somewhat different molecular mechanism of action. Consequently, they were left out from further data analysis. LE values of all remaining agents were fitted to the exponential function together as depicted in Figure 3. The equation $LE=1.0447e^{3.0925c_{M}}$ was obtained with *R*^2^ of 0.948, indicating that the exponential function describes the experimental data very well.

The extension of the bacterial lag phase with increased concentrations of antimicrobial agents has been already reported in several papers [35,36,37]. Unsurprisingly, in the majority of these studies, the compounds of interest were antibiotics. However, the extended lag phases and reduced growth rates of *Streptococcus* have already been observed when adding different tannins (like tannic acid) [37]. However, the exponential extension of the lag phase with the increase in tannin concentration was reported in this study for the first time. This is very likely due to the nature of the method of MIC values determination. Usually, a twofold dilution series of the antimicrobial agent is prepared and, therefore, the exponential trend could not be observed, while in our study, the experimental points were denser and, therefore, the exponential trend was better manifested.

As reported in Table 1, even at the lowest antimicrobial agent concentrations the lag phases were longer compared to the negative control but at agent concentrations right below the MIC they became significantly extended by multiple times compared to the control. The most pronounced extension of the lag time was observed in the case of quebracho extract (21-fold), Colistizer (19-fold), tannic acid (18.6-fold), castalagin (16.8-fold), mimosa extract (16.7-fold), and vescalagin (12.9-fold). The increasing trend was the least noticeable in the case of chestnut and gallic acid. These differences can be at least partly explained by the different $c_{M}$ values, as well as by experimental uncertainty in some cases, however, the variability in the sample composition may also exert a significant effect on the results.

Certain samples, e.g., gallic acid, vescalagin, and castalagin, had high experimental uncertainty, probably since the final values were averaged over numerous repetitions and not all runs provided identical MICs. Consequently, the lag phases were also of varying lengths, since it was observed that they greatly extend above half the MIC. It would, therefore, probably be easier to compare lag times of only a single repetition, instead of six repetitions.

Lengthening of the lag phase or LE represents a mechanism of bacteria with which they respond to the stress caused by antimicrobial agents offering them a survival advantage. It allows bacteria to survive under higher antimicrobial agent concentrations. The antimicrobial agent concentration is high enough to cause stress to the bacteria, but still low enough that it does not fully inhibit bacterial growth. In the study of the influence of antibiotics on bacterial growth, it was established [35,36] that the lag phase extension represents a mechanism of bacteria, which makes their regrowth possible upon removing antimicrobial agents. The general trend is that the longer the lag phase, the more bacteria can survive and regrow when suitable conditions re-emerge. The lag phase extension also enables bacteria to develop the resistance against antimicrobial compounds.

In the lag phase, bacteria synthesize the enzymes and uptake the essential nutrients like different ions (Fe^2+^, Mn^2+^, and Ca^2+^) necessary for the cell growth and division [15]. One of the proposed molecular mechanisms of antibacterial activity of tannins represents their chelation of metal ions [33] and, therefore, the extension of the lag phase could be explained by the formation of coordination compounds between tannins and ions present in the growth medium. With increased concentrations of tannins, an increasing fraction of ions (e.g., Fe^2+^ or Fe^3+)^ is chelated by tannins into coordination compounds and, therefore, inaccessible to the bacteria. The bacteria therefore require an additional time to synthesize an increasing amount of siderophores [39,40] in order to obtain enough essential ions to enable bacterial growth [41]. Tannins hinder bacterial growth via several pathways, and therefore, it is not surprising that several mechanisms of bacterial resistance against tannins are proposed. Next to depletion of essential metal ions, tannins can also inhibit bacterial growth by interacting with cell walls, membranes, or extracellular proteins. However, all these mechanisms require an additional adaptation of bacterial metabolism before bacterial cells can successfully grow in the presence of tannins. As it can be deduced from the exponential dependence of the lag phase extension on $c_{M}$ the higher the concentration of tannins, the longer it takes for the bacteria to successfully adapt to the new environment. Last but not least, data obtained in our study also indicate that tannins can hinder bacterial growth even at the concentrations lower than the MIC.

## 3. Materials and Methods

### 3.1. Studied Agents

Tested samples were vescalagin, castalagin, gallic acid (Sigma-Aldrich, St. Louis, MO, USA), tannic acid (Sigma-Aldrich, St. Louis, MO, USA, 96311-250G-F, Lot: BCBT8361), Colistizer (sample containing tannic acid; Guangzhou Insighter, Guangzhou, Guangdong, China), as well as chestnut (Farmatan, Tanin Sevnica, Sevnica, Slovenia), quebracho (Tannino Red Plus Polvere sacco, Tecnofood, Begoglio, Italy), and mimosa (Tannino Codice M, Tecnofood, Begoglio, Italy) extracts. All purchased compounds were used as received. Castalagin and vescalagin of chromatographic purity 95.9% and 97.0%, respectively, were isolated from the chestnut extract using the procedure described in our previous study [33].

### 3.2. Bacterial Strain

Tannin compounds and tannin extracts were tested against Gram-negative bacteria *Escherichia coli* K12 (DSM 498, Leibniz Institute, DSMZ-German Collection of Microorganisms and Cell Cultures GmbH, Brunswick, Germany). Bacteria were precultured in the nutrient broth composed of 5 g of meat peptone and 3 g of meat extract in 1 L of deionized water (pH was adjusted to 7.0) for 3–4 h in a rotary shaker at 37 °C. Using a Carry 50 UV-Vis spectrophotometer, the turbidity of broth culture was then adjusted to the concentration of 1 × 10^8^ CFU/mL and diluted so that the final desired inoculum concentration on the microtiter plate was of the order of magnitude of 1 × 10^5^ CFU/mL. Bacterial concentration was verified with the spread-plate technique.

### 3.3. Kinetic Growth Assay

The broth microdilution method was performed in 96-well microplates and optical density (OD) was measured using a Tecan Infinite F200 spectrophotometer. Tested samples were dissolved in a growth medium and serially diluted in microplate wells to which the bacterial culture was also added. Growth control of bacteria was performed. Microplates were incubated in a spectrophotometer at 37 °C for 20 h. All OD measurements were performed at a wavelength of 595 nm. The temperature was kept constant between 36.5 and 37.5 °C, the optimal temperature for *E. coli* growth, throughout the experiment. The ODs were recorded every 5 min. Prior to every measurement, 10 s of stirring with an amplitude of 2 mm were applied. For every measurement, an average value of 15 quantifications was used [42]. Lag phases and generation times were reported as the average of six independent measurements.

As already presented in our previous study [33], growth curves, where OD was plotted against time, were drawn and the lowest concentration of tannins, where no bacterial growth was detected, was determined as MIC.

### 3.4. Determination of Lag Times, Bacterial Growth Rates and Generation Times

Generation times (*t*_d_) or doubling times of the bacterial population were obtained so that semilogarithmic graphs of the dependence of OD (in logarithmic scale) on time were drawn. In the exponential growth phase, this dependence of the natural logarithm of OD on time is linear. From the fit of the line to the experimental data in this linear dependence region, we obtained the equation of the line whose slope represents the growth rate (µ) [38]. From it, the generation time was calculated according to Equation (3).

(3) $$\begin{array}{c} t_{d}=\frac{\ln2}{µ} \end{array}$$

The lag time was determined on graphs of the time dependence of OD as the time at which the extrapolated slope of the exponential phase intercepts a horizontal line, extrapolated from the starting inoculum concentration [43].

For a direct comparison of the effect of antimicrobial agent concentration on the lag phase duration, lag time extension (*LE*) [36] defined in Equation (4), was introduced:

(4) $$\begin{array}{c} LE=\frac{\lambda_{c}}{\lambda_{0}} \end{array}$$

In Equation (4), *λ_c_* is the lag time of *E. coli* at the concentration *c* of a given antimicrobial agent and *λ*_0_ is the lag time of the control without the agent. Concentrations of antimicrobial agents were normalized with respect to their MIC values, according to Equation (5),

(5) $$\begin{array}{c} c_{M}=\frac{c_{T}}{c_{MIC}} \end{array}$$

where $c_{T}$ is the concentration of the tested sample and $c_{MIC}$ is the corresponding MIC of the agent. Finally, *LE* was plotted as a function of the normalized concentration for the given agent ($c_{M}$).

### 3.5. Statistical Analysis

For growth rates, generation times, and lag phases at each concentration of tested agent, standard deviations (SD) were calculated according to Equation (6) and their values presented in Table 1.

(6) $$\begin{array}{c} SD=\sqrt{\frac{1}{N-1}\overset{N}{\underset{i=1}{\sum }}{\left(x_{i}-x_{MEAN}\right)}^{2}\;} \end{array}$$

where *N* is the number of parallels, $x_{i}$ are values of growth rates, generation times, and lag phases while $x_{MEAN}$ represents the mean value of the corresponding variable. Variables were determined in six parallels.

## 4. Conclusions

Turbidimetric method was carried out to determine the effect of tannins on the growth phases of *E. coli*. It was observed that the addition of tannins generally decreases growth rates and increases generation times and lag times. The lag phase extension was already observed at low tannin concentrations, while the lag phase became significantly prolonged after their concentrations were higher than half the MIC. The obtained results could be very well described by the exponential dependence of the lag time extension on the tannin concentration. To the best of our knowledge, the current study is, therefore, the first to report that the lag phase prolongates exponentially with increasing tannin concentration.

During the lag phase, bacteria synthesize enzymes required for metabolism and accumulate the microelements as well as nutrients needed for energy or as building blocks in the synthesis of cell structures. Tannins form coordination compounds with several metal ions present in the growth medium, thereby making them unavailable to bacteria. Consequently, a longer time is needed for bacteria to collect a sufficient quantity of microelements and nutrients to initiate cell division.

The results of the present study demonstrate that tannins can control bacterial growth even at concentrations below the MIC by prolonging the lag phase time needed and by extending generation times in the exponential growth phase.

## Figures and Tables

**Table 1:** Growth rates (*µ*), generation times (*t*_d_), and the lag phase durations of all studied antimicrobial agents against *E. coli* with reported standard deviations (SD).

Sample	*c*_sample_ (µg/mL)	µ (min^−1^)	*t*_d_ (min)	Lag (min)
negative control	0	0.043 ± 0.019	20 ± 11	53 ± 9
Chestnut	200	0.030 ± 0.008	25 ± 6	129 ± 19
	300	0.024 ± 0.004	29 ± 5	159 ± 28
	400	0.021 ± 0.004	34 ± 5	205 ± 35
	500	0.025 ± 0.009	30 ± 10	419 ± 126
Quebracho	200	0.024 ± 0.007	30 ± 7	183 ± 39
	300	0.025 ± 0.011	34 ± 18	685 ± 101
	400	0.010 ± 0.001	67 ± 1	1115 ± 10
Colistizer	200	0.034 ± 0.012	22 ± 7	119 ± 14
	300	0.028 ± 0.009	27 ± 8	209 ± 12
	400	0.031 ± 0.013	25 ± 8	307 ± 36
	500	0.032 ± 0.015	27 ± 12	499 ± 86
	600	0.028 ± 0.012	29 ± 12	794 ± 85
	700	0.034 ± 0.025	34 ± 25	1007 ± 128
Mimosa	200	0.045 ± 0.020	17 ± 5	201 ± 35
	300	0.024 ± 0.010	34 ± 13	470 ± 60
	400	0.014 ± 0.007	57 ± 26	886 ± 202
Tannic acid	20	0.026 ± 0.007	28 ± 7	140 ± 2
	30	0.024 ± 0.005	30 ± 6	223 ± 17
	40	0.043 ± 0.020	18 ± 7	655 ± 195
	50	0.009 ± 0.005	89 ± 52	986 ± 10
Gallic acid	300	0.050 ± 0.023	17 ± 8	64 ± 5
	450	0.043 ± 0.016	18 ± 8	85 ± 17
	600	0.031 ± 0.013	27 ± 13	116 ± 36
	750	0.034 ± 0.014	22 ± 7	139 ± 57
	900	0.024 ± 0.009	31 ± 9	280 ± 129
Vescalagin	50	0.038 ± 0.024	22 ± 7	131 ± 11
	100	0.021 ± 0.006	35 ± 11	165 ± 23
	150	0.020 ± 0.005	37 ± 12	206 ± 56
	200	0.017 ± 0.004	45 ± 17	343 ± 127
	250	0.017 ± 0.008	48 ± 23	460 ± 150
	300	0.017 ± 0.006	46 ± 19	684 ± 161
Castalagin	200	0.029 ± 0.005	24 ± 5	175 ± 23
	250	0.016 ± 0.004	45 ± 11	235 ± 46
	300	0.013 ± 0.003	58 ± 23	377 ± 144
	350	0.008 ± 0.003	95 ± 35	691 ± 290
	400	0.005 ± 0.003	199 ± 114	888 ± 279

## Supplementary Materials

The following are available online at https://www.mdpi.com/2223-7747/9/12/1680/s1, Figure S1: Dependence of LE on the $c_{M}$ for all agents, Figures S2–S9: Dependence of LE on the normalized concentration of the chestnut extract (S2), quebracho extract (S3), Colistizer (S4), mimosa extract (S5), tannic acid (S6), gallic acid (S7), vescalagin (S8) and castalagin (S9), Table S1: Exponential functions of LE against $c_{M}$.
