# Supplementary material for: Generation Times of E. coli Prolong with Increasing Tannin Concentration while the Lag Phase Extends Exponentially
Source: Plants (Basel). 2020 Dec 1;9(12):1680. doi: 10.3390/plants9121680 (PMC7760653; doi:10.3390/plants9121680)
Supplement: Supplementary file 1 [file plants-09-01680-s001.pdf]

Plants

Supplementary Material

Generation times of *E. coli* prolong with increasing tannin concentration while the lag phase extends exponentially

*Sara Štumpf [1], Gregor Hostnik [1], Mateja Primožič [1], Maja Leitgeb [1,2], Urban Bren [1,3,]**

[1] Faculty of Chemistry and Chemical Engineering, University of Maribor, 2000 Maribor, Slovenia, [2] Faculty of Medicine, University of Maribor, 2000 Maribor, Slovenia, [3] Faculty of Mathematics, Natural Sciences and Information Technologies, University of Primorska, 6000 Koper, Slovenia

*Corresponding Author

E-mail address: urban.bren@um.si (U. Bren)

Tel.: +386-2-2294-421

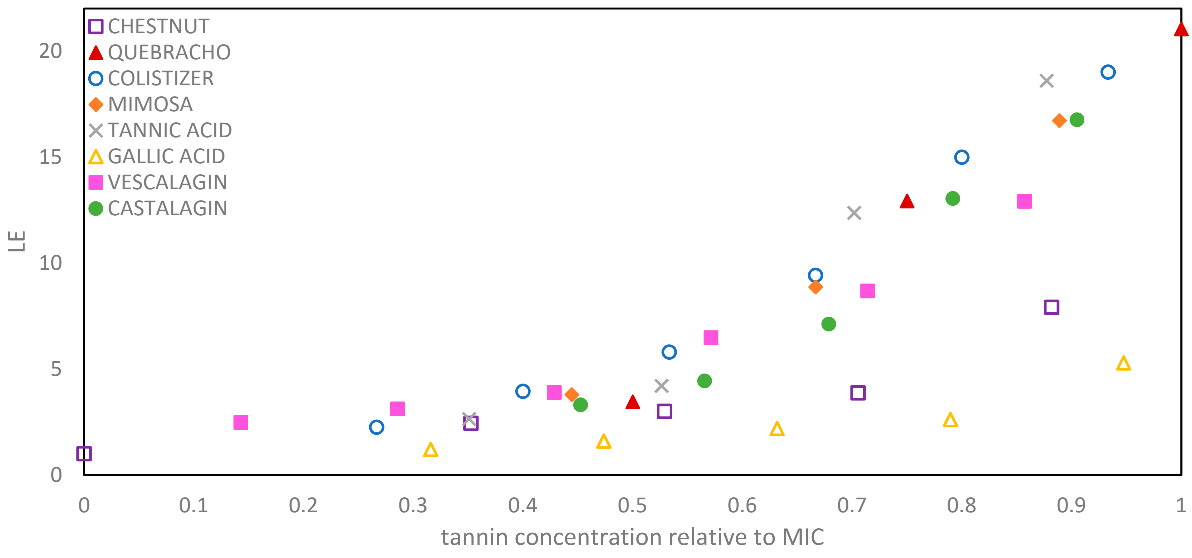

Figure S1. Dependence of lag time extension (LE) on the normalized concentration of investigated agents (c_M).

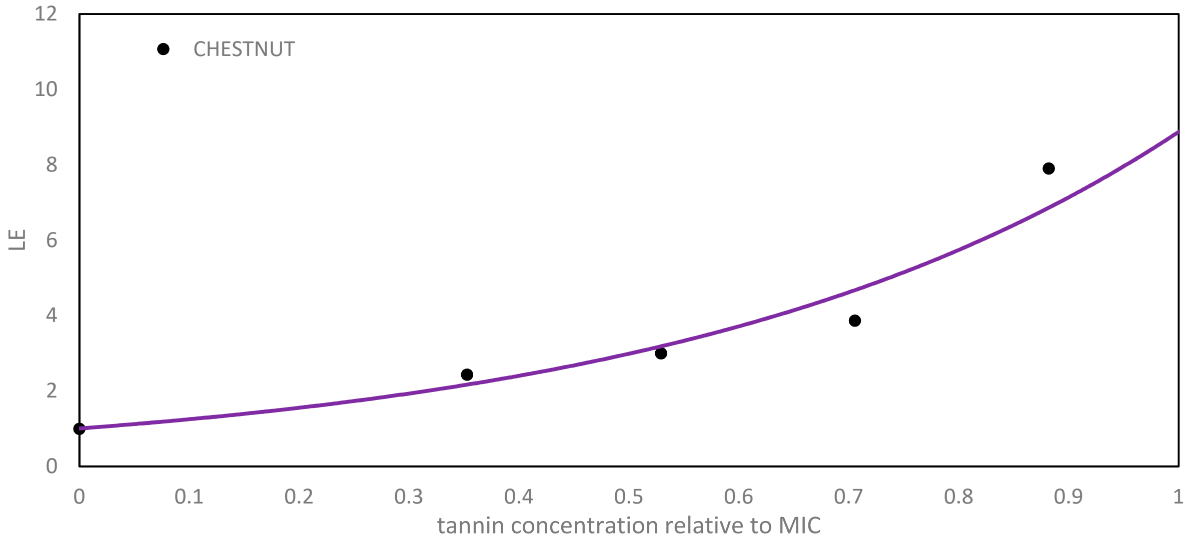

Figure S2. Dependence of lag time extension (LE) on the normalized concentration of the chestnut extract (c_M).

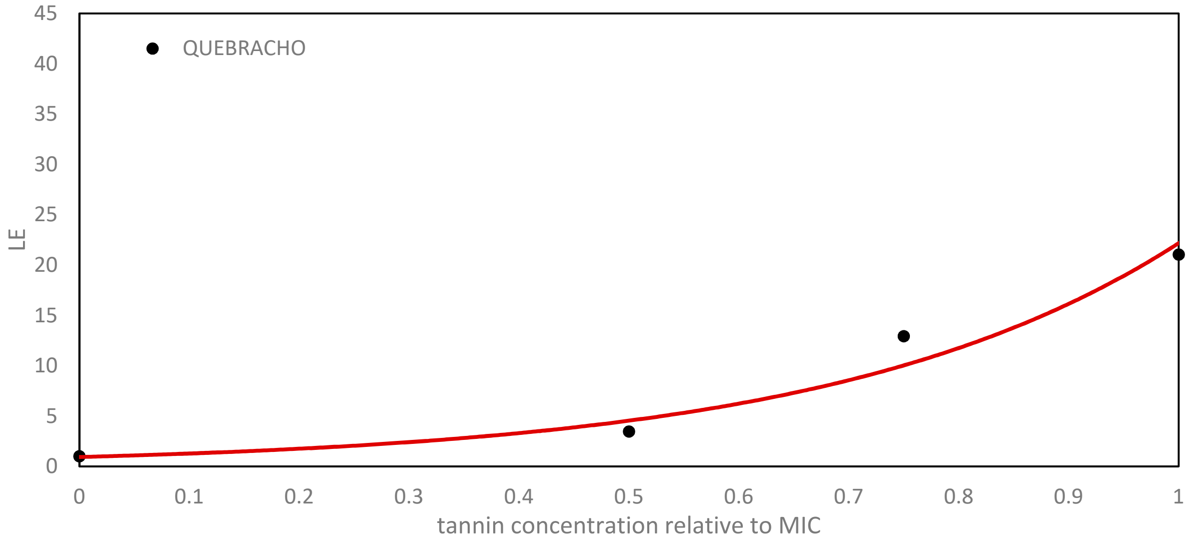

Figure S3. Dependence of lag time extension (LE) on the normalized concentration of the quebracho extract (c_M).

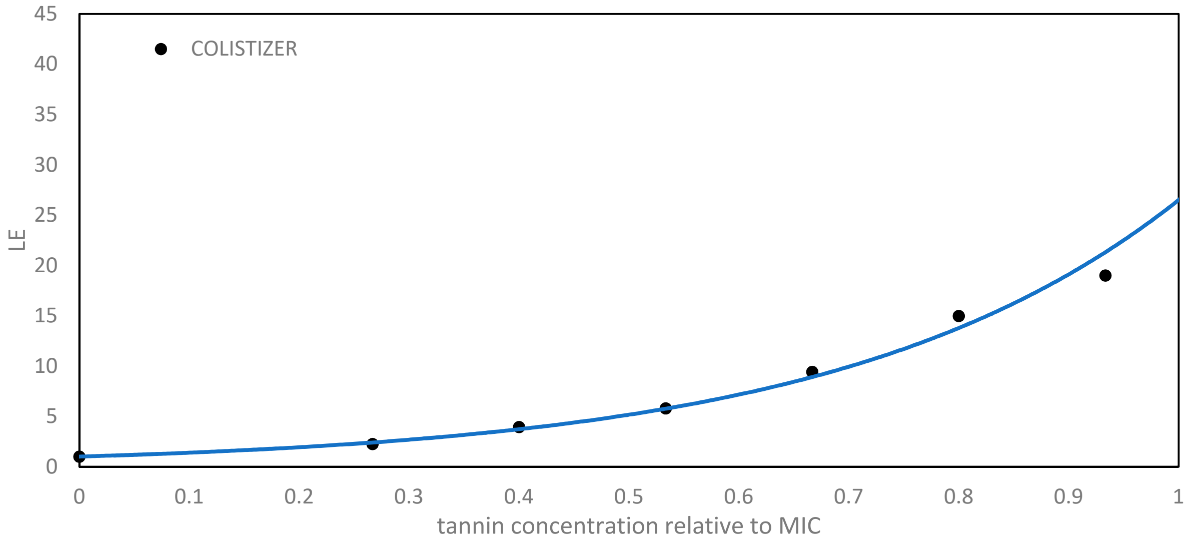

Figure S4. Dependence of lag time extension (LE) on the normalized concentration of Colistizer (c_M).

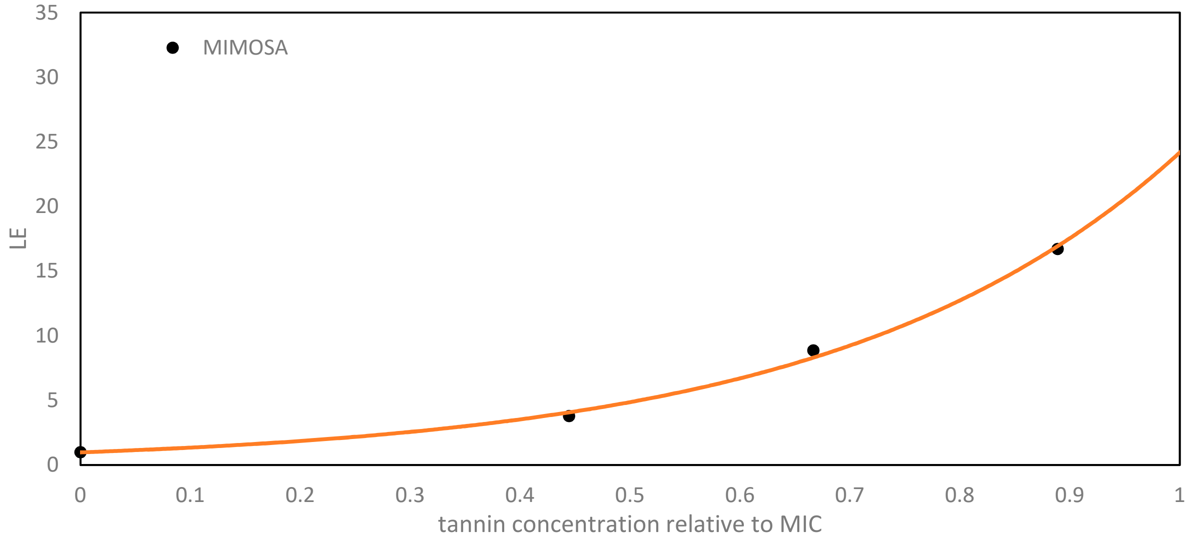

Figure S5. Dependence of lag time extension (LE) on the normalized concentration of the mimosa extract (c_M).

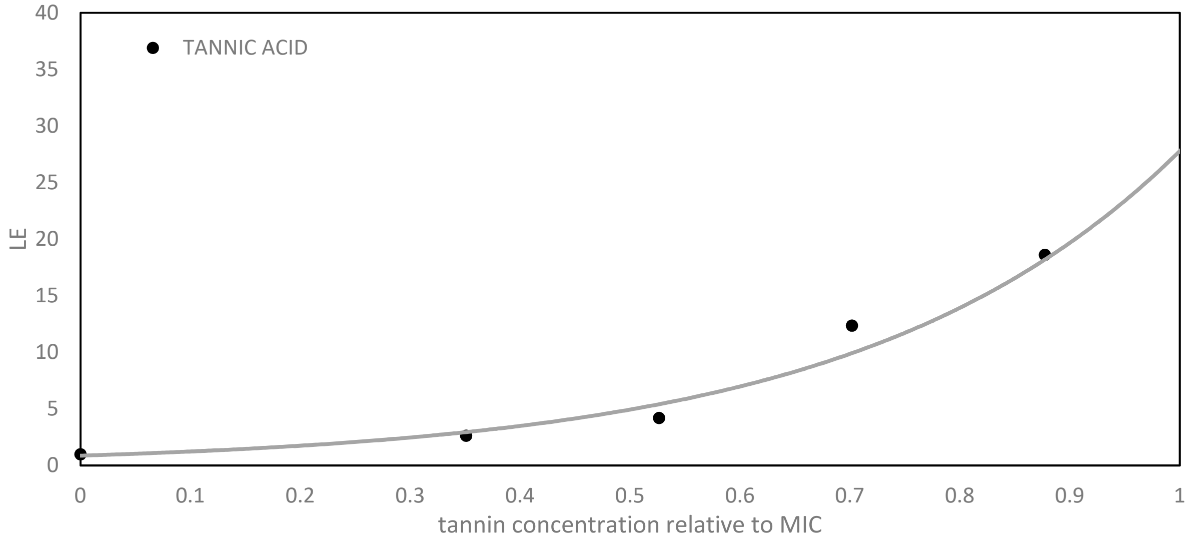

Figure S6. Dependence of lag time extension (LE) on the normalized concentration of tannic acid (c_M).

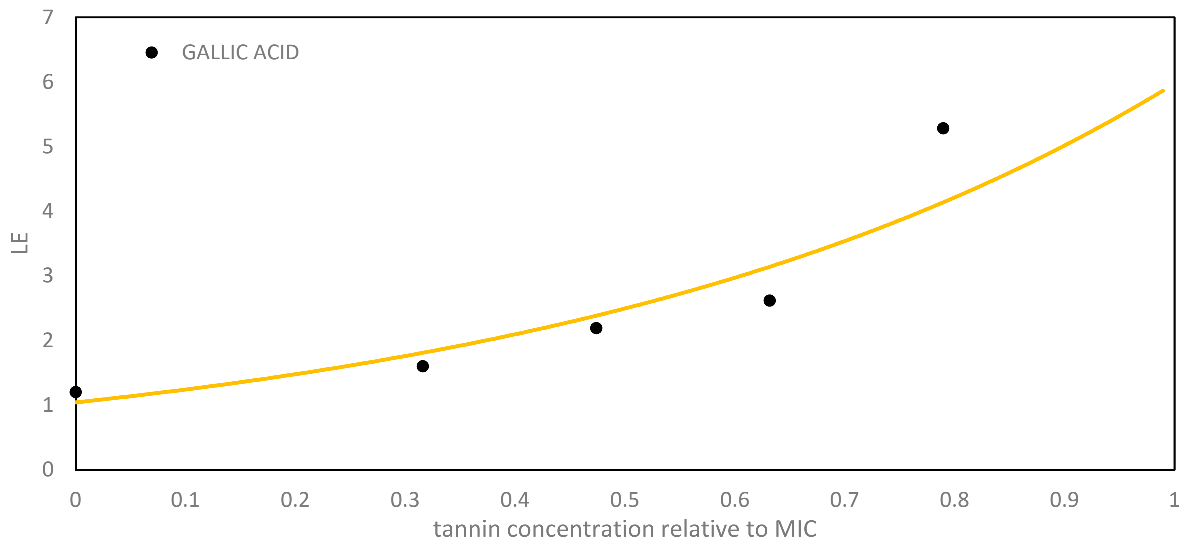

Figure S7. Dependence of lag time extension (LE) on the normalized concentration of gallic acid (c_M).

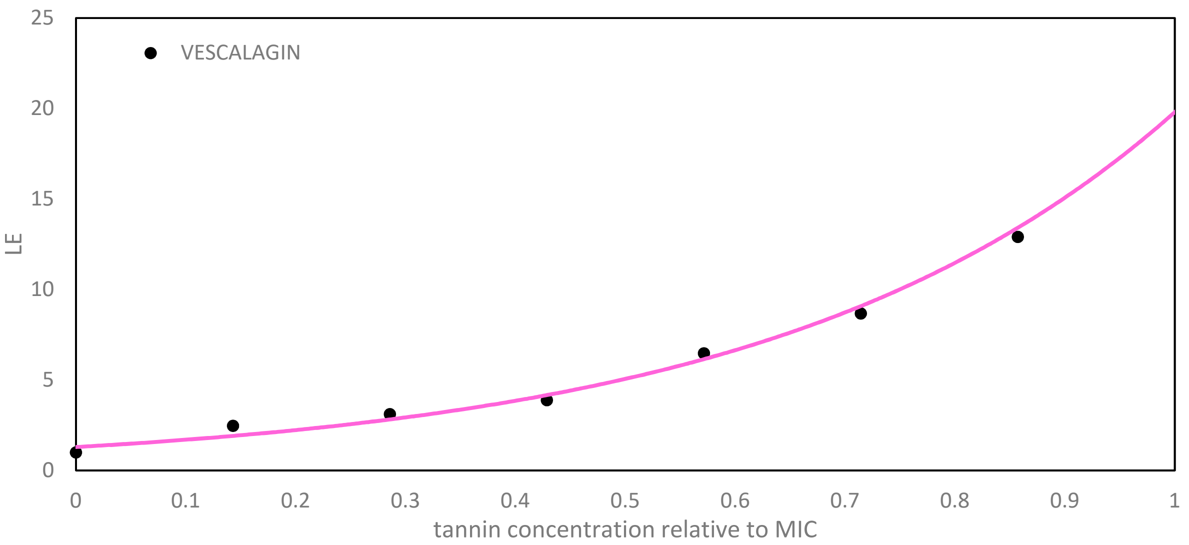

Figure S8. Dependence of lag time extension (LE) on the normalized concentration of vescalagin (c_M).

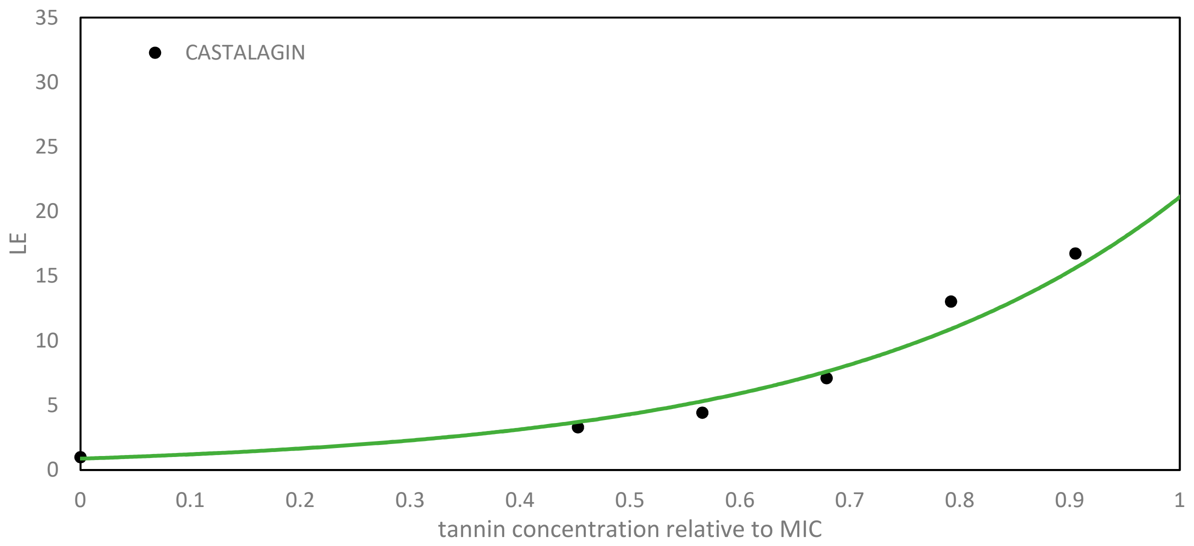

Figure S9. Dependence of lag time extension (LE) on the normalized concentration of castalagin (c_M).

Table S1. Exponential functions of LE against c_M and the corresponding correlation coefficients (R^2).

Sample	LE	R^2
Chestnut	$1.0071e^{2.1761c_M}$	0.968
Quebracho	$0.09291e^{3.1728c_M}$	0.974
Colistizer	$1.0094e^{3.2683c_M}$	0.995
Mimosa	$0.9806e^{3.2052c_M}$	0.998
Tannic acid	$0.8818e^{3.4504c_M}$	0.975
Gallic acid	$1.0431e^{1.7455c_M}$	0.893
Vescalagin	$1.2926e^{2.7292c_M}$	0.965
Castalagin	$0.8844e^{3.1734c_M}$	0.980
